# Immune response induced by novel coronavirus infection

**DOI:** 10.3389/fcimb.2022.988604

**Published:** 2022-10-25

**Authors:** Ying Sun, Yawen Zou, Haiyu Wang, Guangying Cui, Zujiang Yu, Zhigang Ren

**Affiliations:** ^1^ Department of Infectious Diseases, the First Affiliated Hospital of Zhengzhou University, Zhengzhou, China; ^2^ Jinan Microecological Biomedicine Shandong Laboratory, Jinan, China; ^3^ Gene Hospital of Henan Province, Precision Medicine Center, The First Affiliated Hospital of Zhengzhou University, Zhengzhou, China

**Keywords:** SARS-CoV-2, innate immunity, signaling pathways, acquired immunity, CD4+ T cells, CD8+ T cells, immune memory

## Abstract

The coronavirus disease 2019 (COVID-19) pandemic caused by severe acute respiratory syndrome coronavirus (SARS-CoV)-2 has been prominent around the world since it was first discovered, affecting more than 100 million people. Although the symptoms of most infected patients are not serious, there is still a considerable proportion of patients who need hospitalization and even develop fatal symptoms such as cytokine storms, acute respiratory distress syndrome and so on. Cytokine storm is usually described as a collection of clinical manifestations caused by overactivation of the immune system, which plays an important role in tissue injury and multiorgan failure. The immune system of healthy individuals is composed of two interrelated parts, the innate immune system and the adaptive immune system. Innate immunity is the body’s first line of defense against viruses; it can quickly perceive viruses through pattern recognition receptors and activate related inflammatory pathways to clear pathogens. The adaptive immune system is activated by specific antigens and is mainly composed of CD4+ T cells, CD8+ T cells and B cells, which play different roles in viral infection. Here, we discuss the immune response after SARS-CoV-2 infection. In-depth study of the recognition of and response of innate immunity and adaptive immunity to SARS-CoV-2 will help to prevent the development of critical cases and aid the exploration of more targeted treatments.

## Introduction

Novel coronavirus disease 2019 (COVID-19), caused by SARS-CoV-2, was first discovered in Wuhan, Hubei Province, China, in December 2019. It is the pathogen led to the global COVID-19 pandemic. Today, COVID-19 has affected hundreds of millions of people and killed millions, and these numbers are still increasing rapidly. SARS-CoV-2, which can spread rapidly between people, belongs to the coronavirus family and is an enveloped virus with a single-stranded sense RNA genome (Lu et al., 2020). It can invade humans and animals and cause damage to the respiratory system, digestive system, urinary system, nervous system and other systems with varying degrees of severity (Chan et al., 2013; Zumla et al., 2016). The coronavirus family is highly diverse, and six coronaviruses are known to cause disease in humans. Four of them (HCoV-OC43, HCoV-229E, HCoV-NL63 and HCoV-HKU1) bring about only mild respiratory symptoms and have a low incidence of infection (Cheng et al., 2007; Channappanavar et al., 2014; Corman et al., 2019). However, in recent years, two other deadly viruses, SARS-CoV and MERS-CoV, were discovered, infections of which broke out worldwide in 2002 and 2012, respectively, and these viruses pose a serious threat to the lives of infected individuals (Catanzaro et al., 2020). SARS-CoV-2 is the seventh coronavirus discovered after SARS-CoV and MERS-CoV. It is also the third zoonotic coronavirus in the genus Coronavirus, which is transmitted from animals to humans, who serve as intermediate mammalian hosts (Chan et al., 2015; Catanzaro et al., 2020). Similar to other respiratory coronaviruses, SARS-CoV-2 is mainly spread through respiratory droplets and also be spread through the fecal−oral route. Obviously, the prevention, control and treatment of COVID-19 are still very challenging.

The immune system helps the body resist invasion by viruses and other pathogens. The immune system includes the innate immune system and the acquired immune system. Innate immunity is also called nonspecific immunity. The molecular pattern of innate immune cell recognition is called pattern recognition, and the receptors that mediate pattern recognition are called pattern recognition receptors (PRRs). Binding of PRRs to pathogen-associated molecular patterns/damage-associated molecular patterns (PAMPs/DAMPs) promotes cell activation and induces production of inflammatory cytokines, thereby limiting and eliminating viral infection. The acquired immune system, also known as adaptive immunity, can cause the body to respond to specific antigens. It is principally composed of three cell types (CD4+ T cells, CD8+ T cells and B cells) and has the characteristics of specificity, memory and tolerance. Innate immunity is the first line of defense against pathogens, including SARS-CoV-2, and can participate in the initiation and regulation of the adaptive immune response. The acquired immune response and immune memory are the basis of vaccine development, so it is very important to research the immune response to SARS-CoV-2.

## Innate immunity to SARS-CoV-2 infection

Innate immune cells include mononuclear phagocyte system, dendritic cells, neutrophils, natural killer cells (NK cells), innate-like lymphocyte (ILL), mast cells, eosinophils, basophils and so on. After entering the bloodstream, peripheral blood monocytes can pass through vascular endothelial cells to various tissues and organs and develop into mature tissue macrophages. Monocytes can produce IL-1, IL-6, TNF-α, CCLs and other molecules to motivate the release of cytokines and recruit other immune cells (Fajgenbaum and June, 2020). According to clinical statistics, the level of peripheral blood monocytes in healthy controls is lower than that in COVID-19 patients (Wang et al., 2020c; Wang et al., 2020d), and autopsy of COVID-19 patients showed a large amount of macrophage infiltration in the lungs (Bian, 2020; Li et al., 2020). Animal experiments showed that the number of IMM in male mice infected with SARS coronavirus increased by 2-3 times on the third day (Channappanavar et al., 2017). Cytokine analysis showed that the frequency of pro-inflammatory cytokines CCL2 and IL-6 released by male IMM (inflammatory monocyte-macrophages) was higher, so men were more likely to develop acute lung injury and cytokine storm than women. Dendritic cells are key cells in innate immunity and specific immunity *in vivo*, and they are highly functional full-time antigen presenting cells that can stimulate the activation and proliferation of immature T cells, control the inflammatory response, and produce cytokines to regulate immunity. Plasma cell-like dendritic cells are the cells with the strongest ability to produce type I interferon and can express TLR7. TLR7 is a receptor encoded on the X chromosome, which indicates that TLR7 is more expressed in women and produces more interferon-α, which induces more TLR7-mediated immune-related pathways and enhances the host’s ability to eliminate coronavirus (Souyris et al., 2019). During SARS-CoV-2 infection of moDCs *in vitro*, many kinds of proinflammatory chemokines, such as IP-10 and MCP-1, are secreted (Alamri et al., 2021). Studies have shown that there is a correlation between IP-10 and disease severity, and the IP-10 level can predict the progression of the disease (Chi et al., 2020; Yang et al., 2020). Neutrophils are produced in the early stage of infection. They are the most abundant white blood cells in the blood circulation and are also the main component of immune cells. When bacteria, fungi or viruses invade the host, neutrophils can be quickly and efficiently deployed in the infected site (Bardoel et al., 2014; Ley et al., 2018). Together with macrophages, it can phagocytize pathogens and exert bactericidal function, and can also produce cytokines such as TNF- α, ILs, MCSF and chemokines such as IL-8 and CCLs (Cassatella, 1999; Fajgenbaum and June, 2020). According to reports, A low lymphocyte proportion and a high neutrophil/lymphocyte ratio were found to be related to disease severity (Giamarellos-Bourboulis et al., 2020; Kuri-Cervantes et al., 2020; Wu et al., 2020), and the expression of ACE2 was found to be significantly correlated with the activation of neutrophils (Zhou et al., 2019). Severe patients with SARS-CoV-2 infection are often accompanied by neutropenia, and neutrophils related genes such as CD36, GCA and S100P are significantly down-regulated in the recovery period of moderate and severe patients, which may be related to the inhibition of innate immune function in moderate and severe convalescent patients (Zhao et al., 2022). An animal study showed that in the model of male and female mice infected with SARS-CoV, the ratio of neutrophils in bronchoalveolar lavage fluid of male mice was 4-5 times higher than that of female mice 3 days after infection (Channappanavar et al., 2017), and the neutrophils of male rats recruited more CXCL-1 (Sawant et al., 2016). This may be related to the fact that male mice are more prone to lung inflammation, epithelial cell permeability and hemorrhagic damage than female mice. Recent studies have shown that neutrophils can release substances called neutrophil extracellular traps (NETs) to kill microorganisms. When neutrophils are exposed to live SARS-CoV-2 virus, they are more likely to form NET than other neutrophils (Veras et al., 2020). NET is composed of DNA skeleton and contains granulosa proteins such as neutrophil elastase (NE) and myeloperoxidase (MPO) (Thiam et al., 2020). It plays a key role in cytokine storm and thromboregulation after infection with novel coronavirus (Ouwendijk et al., 2021). NETosis is a form of neutrophil death, which participates in the host’s immune defenses by the formation of traps to prevent the pathogen from spreading in the organism (Gillot et al., 2021), but their excessive formation may lead to many negative effects (Caudrillier et al., 2012). In COVID-19 patients, NETosis and NETs may lead to microvascular thrombosis, tissue injury and organ failure (Zuo et al., 2020; Ackermann et al., 2021), and even pulmonary fibrosis, nerve disorder, tumor growth and other post-COVID-19 syndrome (Zhu et al., 2022). NET production has been considered as a predictor to assess disease severity (Didangelos, 2020; Janiuk et al., 2021) and clinical outcomes (Huckriede et al., 2021) in COVID-19. In short, neutrophils showed contradictory activities in pro-inflammatory and anti-inflammatory, antibacterial and autoimmune, and anti-COVID-19 virus-induced immune thrombus regulation. NK cells are a class of lymphocytes that play a key role in antiviral and antitumor responses. According to studies, the diversity of natural killer cell surface receptors is negatively correlated with virus clearance, and NK subsets that express DNAM1 receptors are very important for rapid recovery from SARS-CoV-2 infection (Hsieh et al., 2021). It was found that the number and activity of NK cells in male rodents were higher than those in females (Moulton, 2018). Another study found that during ovulation, NK cells increased with the increase of estrogen and progesterone, but the cytotoxicity of these NK cells may be reduced (Hao et al., 2007). Whether it has any influence on the gender difference of novel coronavirus infection remains to be studied. Mast cells are numerous and widely distributed. It is closely related to the complement system in the process of SARS-CoV-2 infection. In a study of Caucasians, it was found that the levels of C3, C5, C7, C8 and C9 in women were significantly lower than those in men (Gaya da Costa et al., 2018). Complement system has a significant pro-inflammatory response. C3 and C5 proteins can activate mast cell degranulation and trigger cytokine storms (Gralinski et al., 2018). This may be one of the reasons why men are more likely to suffer from cytokine storms than women. In addition, studies have shown that it can also activate a variety of physiological responses, including kallikrein-kinin system (Kunder et al., 2011), which is closely related to vascular high permeability, edema and diffuse alveolar injury in critically ill patients of COVID-19 (Nagashima et al., 2022).

The molecular pattern of innate immune cell recognition is called pattern recognition, and the receptors that mediate pattern recognition are called pattern-recognition receptors (PRRs). PRRs, which exist on the surface of innate immune cells, can recognize pathogen-associated molecular patterns (PAMPs) or damage-associated molecular patterns (DAMPs), which initiates intracellular signal transduction, inflammatory cytokine production and clearance of infected cells. There are five main types of PRRs: Toll-like receptors (TLRs), retinoic acid-inducible gene I (RIG-I)-like receptors (RLRs), nucleotide-binding oligomerization domain (NOD)-like receptors (NLRs), C-type lectin receptors, Z-DNA-binding protein 1 (ZBP1) and absent in melanoma 2 (AIM2)-like receptors (Kanneganti, 2020). To date, it has been confirmed that the activation of TLRs, RLRs, NLRs and inflammatory body-related signaling pathways can occur in response to SARS-CoV-2. These receptors not only mediate innate immunity but also participate in the adaptive immune response and determine the overall health of the immune system.

## The interaction of SARS-CoV-2 with TLRs activates innate immune system pathways

The innate immune system is activated by viruses through TLRs. TLRs are expressed in a variety of immune cells, including dendritic cells, T cells, neutrophils, eosinophils, mast cells, macrophages, monocytes and epithelial cells. Multiple adaptors are used by different TLRs, including MyD88, TIRAP, TRIF and TRAM (Akira and Takeda, 2004). Most TLRs (including TLR1, TLR2, TLR4, TLR5, TLR6, TLR7 and TLR9) can promote the production of inflammatory cytokines through MyD88 signaling (Akira and Takeda, 2004). On the other hand, TLR3 mainly transmits its signal through a MyD88-independent pathway, through TRIF. TLR4 is also unique; it can transmit signals through the MyD88 or TRIF pathways (Diamond and Kanneganti, 2022). Downstream molecules of MyD88, such as nuclear factor (NF)-κB, mitogen-activated protein kinases (MAPKs) and interferon regulatory factors (IRFs), can be activated, which leads to the transcriptional activation of tumor necrosis factor (TNF), interferon-stimulated genes (ISGs) and IFN. TRIF signal transduction can also activate the production of some transcription factors and interferon, thus playing an antiviral role (Akira and Takeda, 2004).

TLR2 mediates inflammation initiated by the SARS-CoV-2 E protein. According to Zheng et al., proinflammatory signaling pathway activity and cytokine production were decreased in TLR2-deficient mouse macrophages. Similarly, the same effects were seen in human macrophages treated with TLR2 inhibitors (Zheng et al., 2021; Potapov et al., 2022). In another animal experiment, K18-hACE2 transgenic mice were injected with TLR2 inhibitors during infection. Two days after infection, the mice treated with TLR2 inhibitors had obviously reduced release of inflammatory factors such as IL-6 and MCP-1, and the survival rate of SARS-CoV-2-infected mice was obviously improved (Zheng et al., 2021). Based on these data, we know that TLR2 can detect SARS-CoV-2 infection *in vivo* and that inhibiting TLR2 signaling can reduce mortality after SARS-CoV-2 infection. The TLR3 signaling pathway may protect against coronavirus infection *in vivo* (Barnard et al., 2006; Zhao et al., 2012; Totura et al., 2015; Lee et al., 2020). Animal experiments by Zhao et al. have shown that TLR3 agonists can promote respiratory epithelial cells to inhibit virus replication and provide a favorable lung environment for a protective immune response (Zhao et al., 2012). However, to date, the effect of TLR3 in SARS-CoV-2 infection is unknown. TLR7 and TLR8 can cooperate with antiphospholipid antibodies to induce the production and release of inflammatory cytokines (Hurst et al., 2009; Doring et al., 2010), and their expression is also upregulated in severe COVID-19 (Borghi et al., 2020; Amezcua-Guerra et al., 2021). Recently, it has been found that the functional deletion variation of TLR7 on X chromosome is related to the pathogenicity of SARS-CoV-2 in young patients (van der Made et al., 2020; Asano et al., 2021), which not only indicates that TLR7 may protect against SARS-CoV-2 infection, but also may be one of the reasons for the gender difference in host immune response to coronavirus. The role of other TLRs in SARS-CoV-2 infection remains to be further studied.

## RLRs recognize SARS-CoV-2 and regulate the interferon pathway

As RNA pattern recognition receptors, RLRs recognize single-stranded RNA from SARS-CoV-2 replication intermediates, and RLRs include MDA5, RIG-I and LGP2 (Rebendenne et al., 2021; Thorne et al., 2021a; Yang et al., 2021; Yin et al., 2021). In particular, MDA5 and RIG-1 play a pivotal role in regulating the production of interferon and the interferon pathway (Yin et al., 2021). Following posttranslational modifications and activation, RIG-1 and MDA5 interact with the adaptor protein mitochondrial antiviral signaling (MAVS) in mitochondria to form the MAVS signalosome. This complex can activate key transcription factors, such as TRAF3, TbK1 and IKK, induce IRF3 phosphorylation (Loo and Gale, 2011), and promote type I and type III IFN gene transcription. After IFN is produced, it binds to its related receptors and stimulates downstream signals through different signal conversion pathways, thus activating a large number of ISGs to produce an antiviral effect (Stark and Darnell, 2012; Wack et al., 2015). Yin et al. used MDA5 and LGP2, as pivotal regulators of antiviral type I IFN production, to study the molecular induction mechanism of interferon in a respiratory epithelial cell line (CALU-3) (Yin et al., 2021). In models of SARS-CoV-2 infection, deletion of MDA5, LGP2 or MAV-related genes leads to a decrease in the expression of type I interferon (Rebendenne et al., 2021; Yang et al., 2021; Yin et al., 2021). It has been confirmed that a highly impaired response to type I interferon seems to be one of the features of severe COVID-19, and the production of inflammatory cytokines is aggravated as a result (Arunachalam et al., 2020; Hadjadj et al., 2020). The role played by RIG-I is still controversial. Silencing the gene encoding RIG-I did not reduce the response of CALU-3 cells to interferon-β during SARS-CoV-2 infection (Yin et al., 2021). However, another study showed that silencing the gene encoding RIG-I significantly reduced the interferon-β response in CALU-3 cells during SARS-CoV-2 infection (Thorne et al., 2021b). The RIG-I helicase domain recognizes the 3’ untranslated region of the SARS-CoV-2 RNA genome but does not recognize the C-terminal domain (Yamada et al., 2021). This unique pattern does not stimulate ATPases and has reduced interaction with MAVS, which ultimately reduces the production of interferon (Kowalinski et al., 2011; Goubau et al., 2013; Takaoka and Yamada, 2019). It is certain that RIG-I plays a unique role in human lung cells as an inhibitor in the early stage of SARS-CoV-2 infection (Yamada et al., 2021).

## NLRs perceive SARS-CoV-2 infection and induce proinflammatory cytokine production

Activation of NLR-related signaling pathways can also produce responses to SARS-CoV-2 infection and induce the production of type I IFNs and proinflammatory cytokines. As one of the most characteristic inflammatory receptors, NLRP3 can be activated by a variety of PAMPs and DAMPs of SARS-CoV, including ORF3a (Siu et al., 2019), ORF8b (Shi et al., 2019), viral RNA (Campbell et al., 2021) and the E protein (Nieto-Torres et al., 2015). After recognizing PAMPs and DAMPs, NLRP3 activates caspase-1, induces the maturation and secretion of bioactive IL-1β and IL-18, cleaves gasdermin (GSDM) D, and ultimately leads to pyroptosis mediated by ninjurin 1 (Christgen and Kanneganti, 2020; Kayagaki et al., 2021). Researchers have found that the severity of COVID-19 is positively correlated with the levels of IL-1β and IL18 in plasma (Laing et al., 2020; Qin et al., 2020). Rodrigues et al., 2021 found NLRP3 and apoptosis-associated speck-like protein containing caspase activation and recruitment domain (ASC) puncta in monocytes and lung tissues of moderate and severe COVID-19 patients, which proved that NLRP3 inflammatory bodies are activated after SARS-CoV-2 infection and are present in COVID-19 patients (Rodrigues et al., 2021).

Moreover, some PAMPs of SARS-CoV-2 can affect NLRP3 inflammasome assembly and cytokine release (Diamond and Kanneganti, 2022). The N protein and ORF3a mentioned above can activate NLRP3 inflammatory bodies in human cell lines (Pan et al., 2021; Xu et al., 2022). TLR2-dependent inflammation can be induced by the E protein of SARS-CoV-2, which may provide activation and activation signals for NLRP3 inflammasome assembly (Nieto-Torres et al., 2015), thus promoting the release of proinflammatory cytokines such as IL-1β to deal with infection (Zheng et al., 2021). In addition, the S protein can drive the formation of NLRP3 inflammatory bodies in macrophages of COVID-19 patients and induce them to secrete mature IL-1β, but this does not occur in macrophages of healthy volunteers (Theobald et al., 2021). AIM2 can perceive host genomic and/or mitochondrial DNA. ASC spots were colocalized with AIM2 and NLRP3 spots in almost all monocytes of COVID-19 patients, but their function is not clear (Junqueira et al., 2021). In addition, NOD1 can regulate the innate immune response and cytokine production after SARS-CoV-2 infection. It has been confirmed that in the context of SARS-CoV-2 infection, the loss of NOD1 in lung epithelial cells can significantly reduce the expression of interferon-β (Yin et al., 2021). However, the role of other inflammatory body sensors in SARS-CoV-2 infection remains to be further studied.

## The CGAS-STING signaling pathway perceives SARS-CoV-2 infection and limits virus replication

The cytoplasmic biosensor cGAS (cyclic GMP-AMP synthase) can also sense viral infection and activate proinflammatory signaling pathways. cGAS-STING (stimulator of interferon genes) plays an important role in limiting virus replication (Schoggins et al., 2014; Sun et al., 2017; Franz et al., 2018). After SARS-CoV-2-induced mitochondrial damage, cGAS is activated by sensing mitochondrial DNA released into the cytoplasm, which mediates the innate immune response (Singh et al., 2020). It has been found that the SARS-CoV-2 helper proteins ORF3a and 3CL can induce evasion of the host innate immune response by interfering with the cGAS-STING signal transduction pathway (Rui et al., 2021). In animal experiments, Li et al. found that exogenous STING agonists can quickly stimulate interferon signals and effectively inhibit infection with many different strains of SARS-CoV-2 to improve the survival rate of transgenic mice (Li et al., 2021).

## Cytokine storms—An imbalance of proinflammatory cytokines after SARS-CoV-2 infection

**Figure 1:**
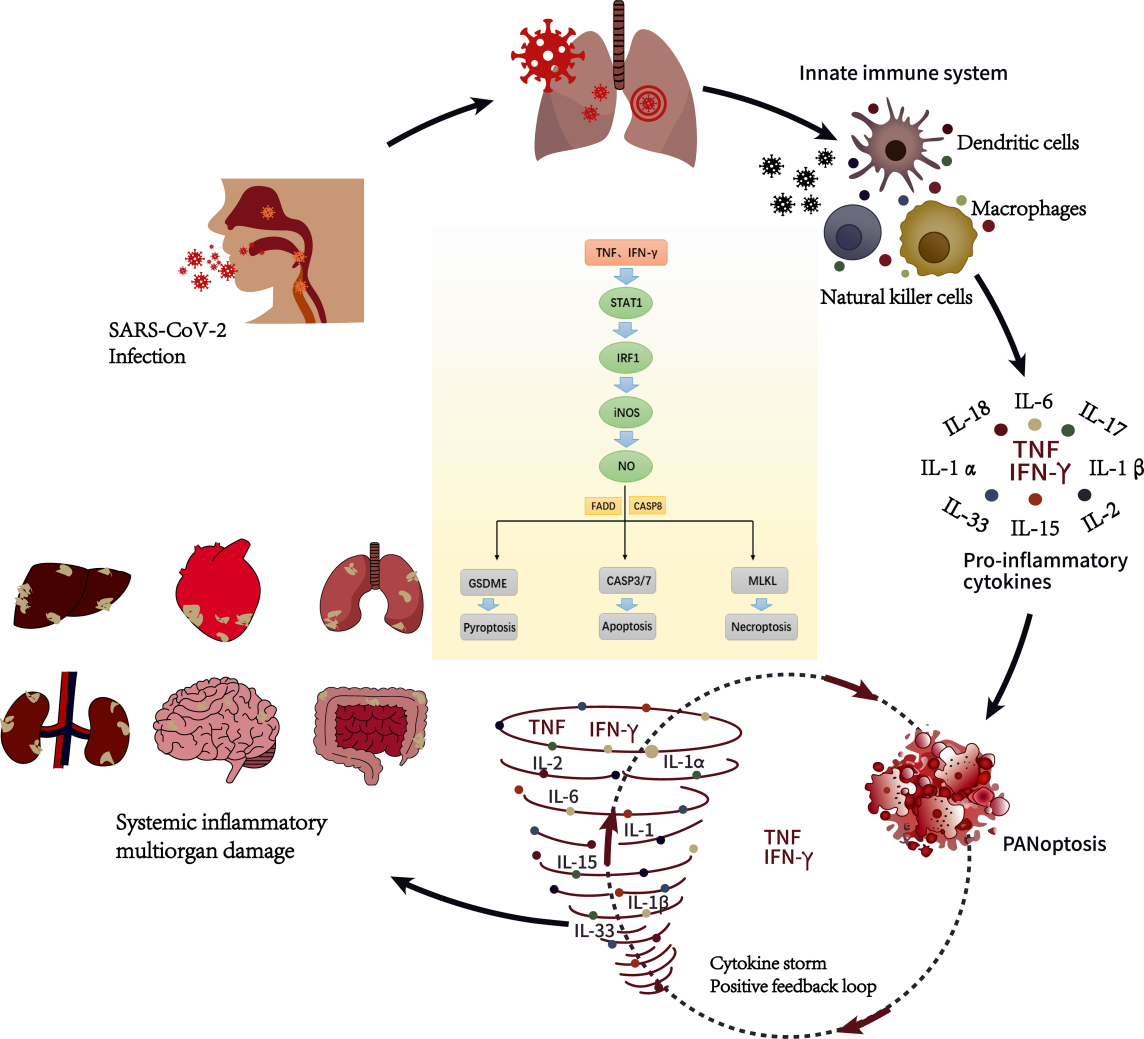
Cytokine storm during SARS-CoV-2 infection (Diamond and Kanneganti, 2022). SARS-CoV-2 enters the host through the respiratory tract. After entering the cells, they will replicate and invade the adjacent cells and extracellular space, of which respiratory symptoms are the most common. After the innate immune system is activated, innate immune cells such as dendritic cells, macrophages and natural killer cells react and release pro-inflammatory cytokines such as TNF and IFN- γ to control infection. When the release of proinflammatory cytokines is unbalanced, it will cause inflammatory cell death characterized by pyroptosis, apoptosis, and necroptosis (PANoptosis). It is characterized by a sharp increase in cytokines and a positive feedback loop in the pathway of programmed cell death related to inflammation, which we call cytokine storm, which can eventually develop into multiple organ dysfunction. Under the joint action of TNF and IFN- γ, it can activate JAK-STAT1-IRF1 axis, induce nitric oxide production and drive caspase8/FADD-mediated PANoptosis.

SARS-CoV-2 invades the body and induces antiviral responses such as immune system activation and the release of inflammatory cytokines. The unrestricted activation of leukocytes also produces a large number of proinflammatory cytokines, including IL-1, IL-6, IFN-γ, IL-18, accompanied by the release of PMN-Net. (Tisoncik et al., 2012). These cytokines not only help the body inhibit and eliminate pathogenic microorganisms but also participate in maintaining the stability of the intracellular environment (Diamond and Kanneganti, 2022). But at the same time, these pathological factors can cause serious damage to inflamed blood vessels and infiltrating tissue, especially in patients with diabetes and hypertension (Tomar et al., 2020). At present, it is believed that there is a positive feedback loop between cytokine release and the cell death pathway (Karki and Kanneganti, 2021). Immune system overactivation leads to a sharp increase in the level of inflammatory cytokines in the circulation, which in turn leads to the release of more cytokines. Finally, a deadly “cytokine storm” is set off (Tisoncik et al., 2012) (**Figure 1**). The inflammatory-related programmed cell death pathways mediated by a large number of cytokines are pyroptosis, apoptosis, and necroptosis (PANoptosis) (Karki and Kanneganti, 2021). In this context, tumor necrosis factor-α and interferon-γ promote Caspase8/FADD-mediated PANoptosis by activating JAK-STAT1-RF1 axis-induced nitric oxide (Karki et al., 2021), which is dependent on PANoptosomes, The caspase (S) complex in the presence or absence of inflammatory components, and RHIM-containing proteins (Diamond and Kanneganti, 2022). Karki et al. reported that tumor necrosis factor-α and interferon-γ can cause inflammatory cell death in mice, which is similar to the cytokine storm observed in critically ill COVID-19 patients. Treatment of mice with neutralizing antibodies against tumor necrosis factor-α and interferon-γ reduced the mortality of cytokine shock in mice infected with SARS-CoV-2 (Karki et al., 2021).

Several studies have analyzed the cytokine spectrum of severe COVID-19 patients and confirmed that there is a direct correlation between inflammatory factor storms and factors associated with poor prognosis, such as lung injury, systemic inflammation, and multiple organ failure (Lucas et al., 2020). Especially in the lung, destruction of endothelial cell membrane structure and vascular injury lead to the occurrence and aggravation of acute respiratory distress syndrome (ARDS) (Berlin et al., 2020; Gandhi et al., 2020). These inflammatory mediators can regulate the function of neutrophils and make them infiltrate into inflammatory foci, and the signal circulation between macrophages and neutrophils is significantly enhanced under the action of cytokine storm. this may lead to persistent inflammation in patients with severe COVID-19 (Akhmerov and Marbán, 2020). Based on this, neutropenia is considered to be an indicator of severe respiratory symptoms and poor prognosis in COVID-19 patients (Guan et al., 2020a; Wang et al., 2020b). The clinical markers related to neutrophils, NLR (neutrophil/lymphocyte ratio) (Liu et al., 2020) and NAR (neutrophil/albumin ratio) (Wang et al., 2020a), are considered to be predictors of mortality in COVID-19 patients, and the increase in their values helps clinicians to identify patients who need more close monitoring and care. DNA, histone, neutrophil elastase (NE), myeloperoxidase (MPO) and cathepsin G released during NETosis have cytotoxic effects on endothelial cells and pulmonary epithelial cells, which can not only enhance the coagulation of tissue factor and coagulation factor XII, lead to intravascular thrombosis and cause multiple organ failure (Massberg et al., 2010; Gould et al., 2014), but also further promote neutrophil recruitment. Promote the occurrence of ARDS and cytokine storm. Cytokine storms in SARS-CoV-2 infection and blood clot formation are closely related to superoxide free radicals and hydrogen peroxide, which are caused by activated neutrophils by triggering respiratory bursts (Barnes et al., 2020; Cecchini and Cecchini, 2020; Laforge et al., 2020).

It has been reported that some severe COVID-19 patients have thromboembolism (Leentjens et al., 2021; Poor, 2021), which is also one of the reasons for COVID-19 ‘s high mortality. The reason may be related to the anticoagulant pathway related to the damage of proinflammatory cytokines to endothelial cells (Esmon, 2005), in which excessive Net plays an important role. Bradykinin, a member of the kallikrein-kinin system (KKS), acts as a vasodilator and inflammatory mediator in various signal cascades, mediating a variety of vascular permeability functions such as thrombosis and coagulation (Costa-Neto et al., 2008). Signal transduction is induced by coupling the transmembrane receptors of kinetin B1 and B2 with different subunits of G protein (Rex et al., 2022). The formation and regulation of bradykinin is initiated by the activation of coagulation factor XII (Alfaro et al., 2022). The signal mechanism mediated by bradykinin can activate a large number of proinflammatory cytokines, including IL-6, IL-1 β, IL-8 and IL-2. It is reported that the expression of BDKRB1 and BDKRB2 receptors in COVID-19 patients is significantly increased, which may even lead to bradykinin storm (Garvin et al., 2020; Kaplan and Ghebrehiwet, 2021). This “bradykinin storm” can lead to vasodilation, changes in vascular permeability and hypotension. At the same time, it is also a potential key trigger for cytokine storm and thromboembolic complications. In rare cases, children develop childhood multisystemic inflammatory syndrome (MISC) 1-2 months after infection with SARS-CoV-2, which is similar to Kawasaki disease, with elevated inflammatory markers, fever and multiple organ dysfunction (Schultze and Aschenbrenner, 2021). However, these patients have unique immunological characteristics in circulating cytokine profiles and the composition of the T-cell compartment (Carter et al., 2020; Consiglio et al., 2020; Gruber et al., 2020), which may be related to the injury of endothelial cells caused by tumor necrosis factor and interferon-γ (Diamond and Kanneganti, 2022). It is worth mentioning that some studies have shown that the innate immune response of convalescent patients with severe COVID-19 has been weakened, which may also be related to the significant down-regulation of genes related to interferon-γ response in several innate cellular subtypes (Zhao et al., 2022).

In addition, when COVID-19 was autopsied, spleen and lymph nodes were depleted of germinal centers (Lax et al., 2020). It may be due to the progressive decrease in lymphocytes caused by tumor necrosis factor and interferon-γ signals during SARS-CoV-2 infection (Bhattacharjee and Banerjee, 2020; Guan et al., 2020b; Karki et al., 2021). It was also found that the degree of lymphopenia was closely related to an increase in IL-6 and IL-8 in circulation (Zhang et al., 2020). Kaneko et al. found that early specific blocking of Bcl6+TFH cell differentiation, an increase in Tbet+TH1 cells and abnormal accumulation of extrafollicular TNF-α were closely related to loss of follicles; this effect hindered B-cell affinity maturation and the production of mature antibodies, which explains the limited persistence of the antibody response in coronavirus infection (Kaneko et al., 2020).

In short, although cytokines play an important role in mediating innate immune response and fighting viral infection, their release must be limited. Cytokine storm is related to a variety of pathological factors. Understanding the molecular mechanism behind it is helpful for us to develop more targeted treatment strategies to prevent “cytokine storm” during SARS-CoV-2 infection from threatening the lives of infected people.

## Acquired immunity to SARS-CoV-2 infection

Acquired immunity, also known as adaptive immunity, mainly features virus-specific CD4+ T cells, CD8+ T cells and antibodies. It is the response of the body to a specific antigen that is unique to the individual and is not heritable. When the first line of defense cannot limit the pathogen, adaptive immunity, as the second line of defense, will intervene. Adaptive immunity plays an irreplaceable role in controlling and eliminating viral infection, and the immune memory it produces is the key to the success of vaccines.

## CD4+ T cells

A T-cell response is common after the host is infected by SARS-CoV-2 (Rydyznski Rydyznski Moderbacher et al., 2020). CD4+ T cells are functionally heterogeneous T cells, and the main subset is helper T cells. The molecular complex formed with a TCR recognizes an antigen peptide in the context of MHC class II on the surface of CD4+ T cells can exert effects by inducing the secretion of a variety of cytokines. CD4+ T cells mainly assist B cell activation and production of antibodies, activate CD8+ T cells, activate macrophages and enhance the ability of macrophages to kill intracellular bacteria and their antigen presentation. CD4+ T cells are closely related to the control of primary infection, and the response of CD4+ T cells to SARS-CoV-2 is more significant than that of CD8+ T cells (Grifoni et al., 2020).

**Figure 2:**
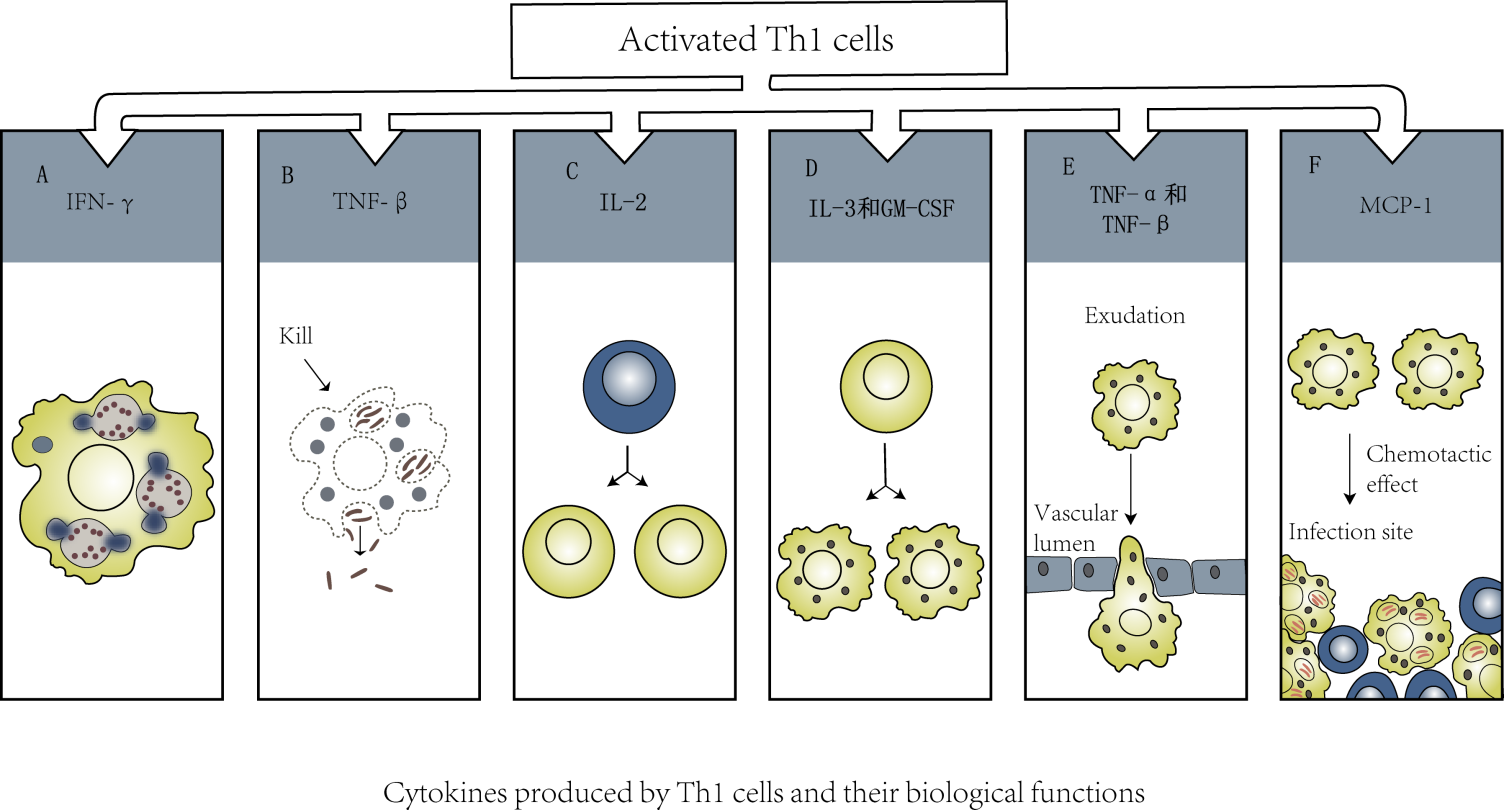
**(A)**. IFN-γ→Activate macrophages and enhance their phagocytosis and killing of intracellular pathogens. **(B)**. TNF-β→Kill macrophages with chronic infection. **(C)**. IL-2→Promote activation and proliferation of T cell subsets. **(D)**. IL-3 and GM-CSF→Promote the differentiation of bone marrow hematopoietic stem cells into macrophages. **(E)**. TNF-α、TNF-β→Promote newborn macrophages to infiltrate infected tissue through blood vessel wall. **(F)**. MCP-1→Recruit macrophages to the infected focus to exert its effect.

T cells that specifically recognize any viral protein may be related to protective immunity. Virus-specific CD4+ T cells can differentiate into Th1 cells and follicular helper T (TFH) cells. Th1 cells can regulate a variety of immune cells by mediating the cellular immune response (**Figure 2**); they can secrete cytokines such as IFN-γ and TNF to promote the cellular immune response and have a direct antiviral function. There are no reports of gender differences between Th1 and COVID-19 infection, but the significance of Th1/Th2 balance is very important for pregnant women infected with SARS-CoV-2. Although pregnancy is reported to be associated with an increased risk of entering ICU and receiving mechanical ventilation, it is not associated with an increased risk of death (Ellington et al., 2020). In order to protect the fetus from excessive immune response, the immune system will undergo the weakening of Th1 response and the conversion of Th2 anti-inflammatory pathway during pregnancy (Dashraath et al., 2020). TFH cells are the key T-cell subset that assists B cells in producing antibodies, and B cells are likely to die within 1 day after antigen recognition without the help of T cells (Akkaya et al., 2018). B cells play a critical role in most neutralizing antibody responses and the development of long-term humoral immunity (Crotty, 2019). Specific circulating TFH (CTFH) cells are produced during acute infection and recovery from SARS-CoV-2 infection. Studies have shown that the frequency of SARS-CoV-2 CTFH cells is positively correlated with the severity of disease (Rydyznski Rydyznski Moderbacher et al., 2020).

Grifoni et al. used T-cell receptor (TCR)-dependent activation induced marker (AIM) assays to identify and quantify SARS-CoV-2-specific CD4+ T cells in recovered COVID-19 patients. The results showed that in terms of the CD4+ T-cell response per donor, the response to the Spike protein was as high as 50% (Grifoni et al., 2020). Therefore, researchers conducted an in-depth study of the SARS-CoV-2 Spike protein, hoping to find a more effective vaccine. According to the experiments of Grifoni et al., the most prominent targets of SARS-CoV-2-specific CD4+ T cells are the Spike, M and Nucleocapsid proteins, and the others, such as ORF3a and Nsp3, are relatively weak to target (Grifoni et al., 2020; Le Bert et al., 2020c; Oja et al., 2020b; Nelde et al., 2021). M is a small protein with multiple transmembrane regions. Although high-affinity class II-restricted T cells specifically recognizing the M protein have not been identified, it is stably recognized by CD4+ T cells in some COVID-19 cases (Grifoni et al., 2020). In addition, mapping of M and S (spike protein) epitopes has shown that the highly expressed proteins during infection can be the first to be reacted by memory CD4+ T cells, which does not depend on the existence of class II epitopes (Mateus et al., 2020b). During acute infection, recovery and memory, the pattern of SARS-CoV-2 antigens recognized by CD4+ T cells may be similar, and CD4+ T cells mediate most of neutralizing antibody responses, persistent antibody response and affinity-matured B cell memory (Rydyznski Moderbacher et al., 2020). Therefore, vaccine research should mainly focus on the immune response of CD4+ T cells.

## CD8+ T cells

CD8+ T cells, also known as cytotoxic T cells, are effector cells with killing activity. The TCRs of these T cells can recognize the antigenic peptide-MHC class I molecular complex on the surface of target cells (such as virus-infected cells and tumor cells). It has been confirmed that the level of SARS-CoV-2-specific CD8+ T cells is closely related to disease outcome (Rydyznski Moderbacher et al., 2020), and in one study, even on the first day after the onset of symptoms, specific CD8+ T cells had begun to expand (these cells were not detected before infection) (Schulien et al., 2021). More importantly, in terms of functional characteristics, SARS-CoV-2-specific memory CD8+ T cells are very similar to influenza-specific CD8+ T cells, even in convalescent patients with negative anti-SARS-CoV-2 antibody (S) and nucleoprotein (N)-seronegative patients (Schulien et al., 2021). These results show that cross-reactivity and specific CD8+ T-cell reactions are important factors of immune protection in mild infection. On the other hand, in the course of infection, the antibody response may weaken faster than the specific CD8+ T-cell response (Cao et al., 2007; Ng et al., 2016). SARS-CoV-2-specific CD8+ T cells can recognize many SARS-CoV-2 antigens, the most prominent of which are the Spike, nucleocapsid, M and ORF3a proteins (Grifoni et al., 2020; Le Bert et al., 2020b; Schulien et al., 2021; Sette and Crotty, 2021). SARS-CoV-2-specific CD8+ T cells mainly express granzyme B, TNF-α, perforin, CD107a and other molecules, which can produce a strong cytotoxic effect (Rydyznski Moderbacher et al., 2020; Sette and Crotty, 2021). In addition, healthy women usually show up-regulated higher cytotoxic T-cell activity and CD8+ genes (Quinti et al., 2020). Women have higher levels of activation of CD8+T cells than men, which may be related to the higher levels of activation of TLR7 and pDC in women (Cervantes-Barragan et al., 2007). Its advantage is that it may reduce the viral load of early novel coronavirus infection. SARS-CoV-2-specific CD4+ T cells and CD8+ T cells play their respective roles in the adaptive immune response, and they coordinate with each other to play a protective role that can have rapid effects during acute COVID-19 infection.

## Plasma cells and antibodies

**Figure 3:**
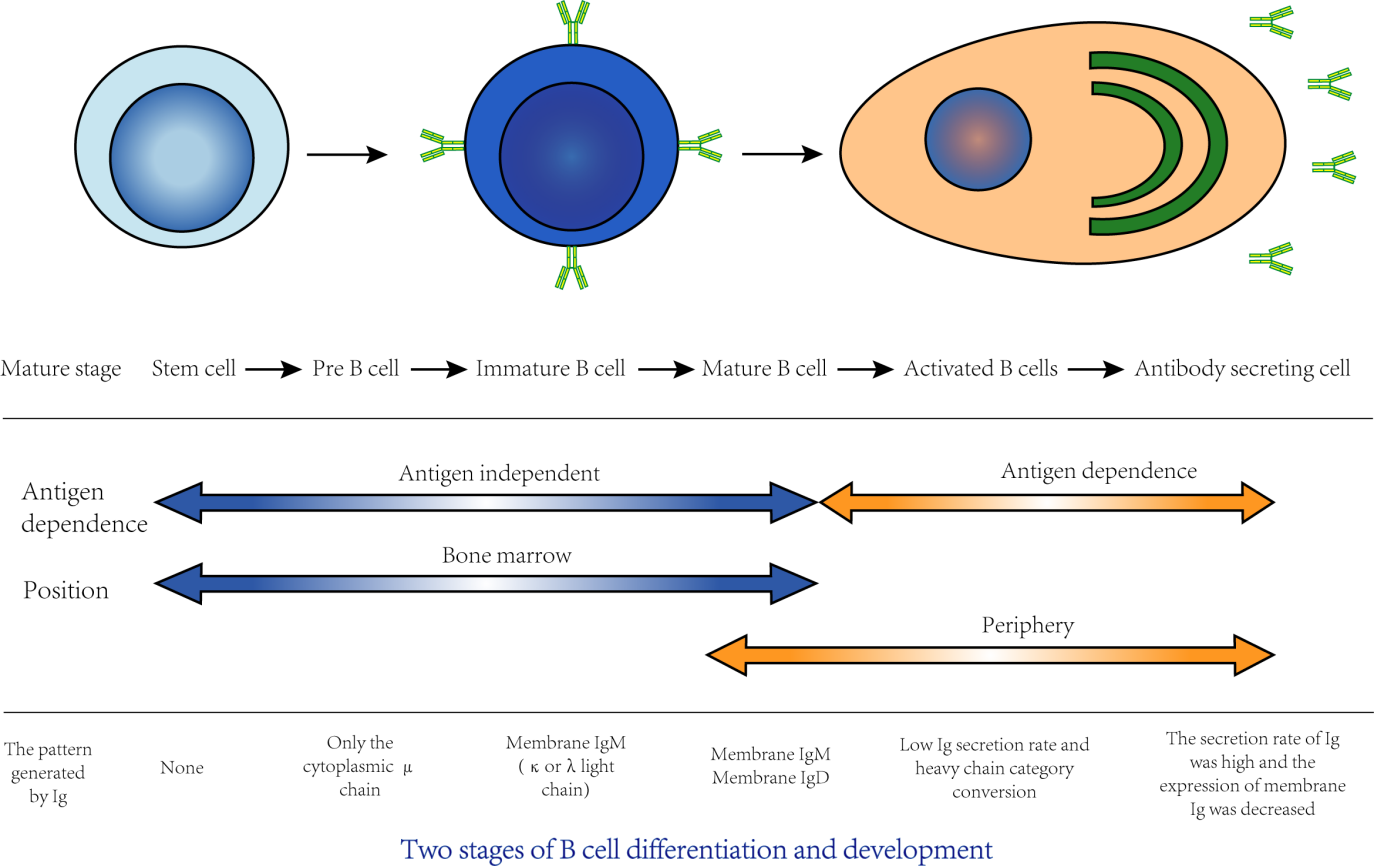
The differentiation and development of B cells can be divided into two stages: central development and peripheral development. Central development: Stem cells in bone marrow go through the stage of pre-B cells and immature B cells and finally develop into mature B cells, which is also known as the antigen-independent stage of B cell development. This process is closely related to bone marrow hematopoietic microenvironment. Cytokines and adhesion factors in bone marrow stroma are key factors involved in development. Peripheral development: Mature B cells migrate to peripheral immune organs. If it is stimulated by antigen, B cells will proliferate, differentiate into plasma cells and secrete specific antibodies, which is also known as the antigen-dependent period of B cell development. Most of these activated B cells differentiate into plasma cells that secrete specific antibodies, and a few differentiate into memory B cells.

B lymphocytes are referred to as B cells, and their development can be divided into central development and peripheral development. In central development, progenitor B cells derived from bone marrow lymphoid stem cells differentiate and develop into mature B cells in bone marrow, while in peripheral development, mature B cells that migrate to peripheral lymphoid tissue differentiate into plasma cells that can produce antibodies after antigen stimulation (**Figure 3**). Most B cells can eventually differentiate into plasma cells that can secrete specific antibodies, and a few B cells can differentiate into long-lived memory B cells. The RBD domain of Spike (S) is an important target of neutralizing antibodies in SARS-CoV-2 (Ju et al., 2020). In a study of Rydyznski et al., RBD IgG was detected in almost all COVID-19 patients, and the level of RBD IgA was strongly correlated with that of RBD IgG. Although RBD IgM is rarely detected, it can be found in both acute and convalescent stages. Other related antibodies, such as S IgG, S IgA, N IgG and N IgA, can also be detected. A series of serological tests found that circulating antibodies against SARS-CoV-2 RBD, S and N, as well as neutralizing antibodies, could be detected in most COVID-19 patients in the acute and convalescent stages (Rydyznski Moderbacher et al., 2020). SARS-CoV-2 nucleocapsid protein (N) and Spike are often used for serum diagnostic analysis. In the reports of Long and others, the large majority of people infected with SARS-CoV-2 had seroconversion within 5-15 days PSO, and almost all infected people had seroconversion at 19 days PSO (Long et al., 2020).

Antibodies are immune proteins produced by human B lymphocytes stimulated by antigens and mainly exist in blood and tissue. When microbes invade the human body, only some antibodies can recognize these microorganisms and capture them. Such antibodies are called neutralizing antibodies. Most of SARS-CoV-2-infected people, the time window for the production of neutralizing antibodies is roughly the same as that for seroconversion, which may be related to the accelerated production of cytokines that promote antibody type conversion (Avery et al., 2008). These interleukins are part of the Th2 pathway, which is naturally enhanced in women, suggesting that there may also be gender differences in antibody conversion (Zhou et al., 2020). It is reported that there is no significant gender difference in serum immunoglobulin levels between mild and convalescent patients, but serum immunoglobulin levels in women increase significantly in the early stage of infection and critical stage of the disease (Zeng et al., 2020). However, it is uncertain whether this titer change has an impact on the prognosis. RBD-bound B cells with a wide range of heavy chain and light chain V genes can produce such neutralizing antibodies (Robbiani et al., 2020). These neutralizing antibodies come not from preexisting cross-reactive B cells but from immature B cells (Anderson et al., 2020). Therefore, the neutralizing epitopes in the SARS-CoV-2 RBD domain have relatively strong immunogenicity and are easily recognized by antibodies. Although neutralizing antibodies have nothing to do with COVID-19 disease remission (Baumgarth et al., 2020; Rydyznski Moderbacher et al., 2020), they are closely related to protection against SARS-CoV-2 secondary infection, which has been confirmed in nonhuman primate models (Chandrashekar et al., 2020; Deng et al., 2020; Gao et al., 2020; Krammer, 2020; Subbarao, 2020). In addition, antibodies can also kill cells infected with the virus. Although this concept has not been confirmed in human infections, it has been found that activating FC receptors in small animal models of SARS-CoV-2 infection makes the protective effect of anti-SARS-CoV-2 antibodies stronger *in vivo* (Schafer et al., 2021). In other words, neutralizing antibodies with FC receptor binding ability are more potent (Sette and Crotty, 2021). Fc-mediated effector function is important in the antiviral response *in vivo* (Piccoli et al., 2020). In large cohort studies, neutralizing antibody titers have been found to be positively correlated with the severity of COVID-19. It has also been confirmed in various animal models that there is a positive correlation between antibody titer and antigen load. However, the connection between neutralizing antibodies and the severity of COVID-19 is very complicated. High and medium antibody titers and the activation of extrafollicular B cells are commonly found in critically ill patients (Woodruff et al., 2020). Control of SARS-CoV-2 infection requires balanced participation of B cells and T cells (Oja et al., 2020a). Impairment of innate immune function and delayed activation of T cells in the effective control of viral load can disrupt this balance, which may produce a disordered immune response and worsened condition of critically ill patients with COVID-19.

## Immune memory

Immune memory of a virus is mainly maintained by four components: memory CD4+ T cells, memory CD8+ T cells, memory B cells and antibodies. The main function of immune memory is to protect against reinfection, and the subtypes of immune memory and local tissue immune memory are very important (Orenstein and Ahmed, 2017; Piot et al., 2019). First, we will discuss immune cross-reactivity. It was proven that T-cell cross-recognition occurs between circulating common cold-causing coronaviruses and SARS-CoV-2. Most SARS-CoV-2 cross-reactive T cells are CD4+ T cells (Grifoni et al., 2020). It has been proven that these cells are memory T cells, many of which are memory T cells from infections with common coronaviruses with conserved epitopes (Mateus et al., 2020a). CD8+ T cells are rarely found (Grifoni et al., 2020). However, these findings are enough to show that some levels of preexisting immunity in the population can provide some protective immunity against respiratory tract virus infections (Sette and Crotty, 2020). Epidemiological evidence from large cohort reports controlling for other factors, such as age, infection with HCoV in the past 3 years significantly reduced the risk of severe disease in SARS-CoV-2 patients (Sagar et al., 2021). While memory B cells rarely cross-react in humans, the antibody response to related viruses may also affect protective immunity (Gostic et al., 2016; Aydillo et al., 2021).

Whether immune memory will be induced after SARS-CoV-2 infection cannot be directly predicted. It is necessary to collect data from at least 6 months after infection in order to determine whether immune memory lasts for many years. Because SARS-CoV-2 has been infecting humans for a short time, the protective and pathogenic aspects after infection are still unclear. The study of the immune response to SARS-inducing coronavirus and Middle East respiratory syndrome coronavirus (MERS-CoV) can help us to further understand the immune response to coronavirus. Memory T cells were detected in 23 recovered SARS patients 17 years after the outbreak of SARS in 2003 (Le Bert et al., 2020a). A SARS-CoV Ag-specific memory B-cell response could not be detected after 6 years (Tang et al., 2011), but neutralizing antibodies were detected for at least 17 years (Tan et al., 2020). Studies on MERS are relatively limited. Although the specific antibody response to MERS-CoV persists for at least two years in patients recovering from severe diseases, no or only transient response is detected in patients with subclinical or mild disease, and even those who show a response gradually lose this response within two years after infection (Drosten et al., 2014; Zhao et al., 2017). However, a memory T-cell response can be detected in all patients, including those without an antibody response, indicating that although the antibody response is short, at least some immune memory remains (Zhao et al., 2017). Detecting circulating antibodies is the easiest way to judge immune memory. Two studies of more than 1000 subjects have shown that circulating SARS-CoV-2 IgG titers can be maintained for at least 3-4 months (Gudbjartsson et al., 2020; Wajnberg et al., 2020). Twenty-five percent of patients become seronegative within 6 months (Ward et al., 2020). Because of the potential role of RBD IgG, Spike IgA, RBD-specific memory B cells, SARS-CoV-2-specific CD4+ T cells, and SARS-CoV-2-specific CD8+ T cells in the protective immunity against SARS-CoV-2, these components are often evaluated to determine the quality of immune memory. Most people with COVID-19 have all five immune factors at 1-2 months PSO, and at least 3 of the five immune factors are still present in 95% of infected patients after 5-8 months. In general, immune memory is heterogeneous, with different patterns of immune memory in different individuals (Dan et al., 2020).

The specific IgG antibodies, memory B cells and memory T cells produced in patients with mild COVID-19 can last at least three months (Rodda et al., 2021). Upon reinfection, pathogen-specific memory B cells (MBCs) that express receptors associated with antigen experience and the transcription factor T-bet rapidly proliferate and differentiate into plasmablasts (PBs) (Kim et al., 2019; Knox et al., 2019; Nellore et al., 2019), which can secrete protective IgG antibodies. Reactivated T-bet-expressing memory CD4+ T cells proliferate, help activate MBCs and secrete cytokines to activate innate cells (Ruterbusch et al., 2020). In addition, memory CD8+ T cells also secrete cytokines and kill virus-infected cells directly through the delivery of cytolytic molecules (Schmidt and Varga, 2018). Memory T cells can be classified according to anatomical location and transport pattern. Recirculating central memory T cells (TCM cells) and effector memory T cells (TEM cells) can gather at infected sites in the blood circulation and lymph nodes in response to inflammatory signals (Chang et al., 2014). These memory cells that perennially reside in specific nonlymphoid tissues (such as the lungs and upper respiratory tract) are called tissue-resident memory T cells (TRM cells) and may be involved in the immune response to upper respiratory tract infection and lung infection (Masopust and Soerens, 2019). In contrast, TCM/TEM cells respond more slowly to infection and usually proliferate for a few days before entering the infected tissue (Lipsitch et al., 2020). Memory T cells play an important protective role in COVID-19. In a clinical study of 95 infected people, T- and B-cell memory was evaluated 6 months after infection. It was found that 90% of patients had memory CD4+ T cells, and 70% of patients had memory CD8+ T cells (Zuo et al., 2021). Memory CD4+ T cells were more plentiful than memory CD8+ T cells. Memory CD4+ T cells are mainly composed of Th1 and Tfh cells (Dan et al., 2020; Zuo et al., 2021). A study by Zuo et al. predicted that CD4+ and CD8+ T-cell memory has a half-life of 3-5 months (Akondy et al., 2017) but may reach a more stable plateau 8 months after infection, or the decline in such memory may slow down over time (Dan et al., 2020). Zhao et al. found that the expression of activation genes of regulatory T cells, lymphocytes and leukocytes increased in convalescent patients of COVID-19, the expression of adhesion genes between regulatory cells and cells increased, the expression of suppressor genes of T cell response decreased, and the immune response mediated by T cells did not attenuate or even increase (Zhao et al., 2022).

Several studies have shown that memory B cells specific for Spike, RBD, or nucleocapsid can be detected in subjects more than 6 months after COVID-19 (Dan et al., 2020). Of these, 95% of Spike-specific memory B cells produced IgG, and very few produced IgA (Dan et al., 2020). According to a previous study, IgG+ RBD-specific classical memory B cells can produce and continue to produce immune responses in patients with mild COVID-19, and their number will continue to increase from 1 to 3 months (Rodda et al., 2021). There is no obvious decrease from 5-8 months after infection, and they can express more effective neutralizing antibodies (Gaebler et al., 2021). According to the current data, the memory T cells, memory B cells and antibodies produced by most people infected with SARS-CoV-2 are likely to persist for several years (Dan et al., 2020; Wajnberg et al., 2020; Gaebler et al., 2021; Zuo et al., 2021). Studies have shown that patients recovering from SARS-CoV-2 infection can still show high titers of IgG antibodies 2 weeks after discharge (-2 infection can still show high titers of IgG antibodies 2 weeks after discharge (Ni et al., 2020). Another study showed that the level of (RBD)-specific IgG specific to the receptor domain decreased gradually within 6 to 10 weeks of SARS-CoV-2 infection (Beaudoin-Bussières et al., 2020), indicating that B lymphocytes have dual functions in the convalescent phase. The difference in results may be related to the severity of infection, which still needs further study, but it is certain that natural exposure or infection can prevent the recurrence of severe COVID-19.

## Summary and future directions

The continuous emergence of SARS-CoV-2 mutants with immune escape characteristics is the biggest problem troubling us at present. The SARS-CoV-2RNA genome encodes a series of structural and non-structural proteins (Arya et al., 2021; Voloch et al., 2021), The variation between variants is caused by various molecular changes in protein properties caused by mutations in these proteins (Gralinski and Menachery, 2020). The spike protein mutation of SARS-CoV-2 mutant can significantly affect the conformational structure of spike protein, and then affect the interaction with ACE2 or neutralizing antibody (Kim et al., 2022; Saito et al., 2022). In short, each mutant has a different mutation pattern, and the current research focuses on the effect of mutation on Spike protein, because it plays a key role in receptor binding and antibody evasion (Sun et al., 2022).

At present, preventive vaccine is still the mainstream to prevent SARS-CoV-2 infection. The existing SARS-CoV-2 vaccines mainly include inactivated vaccine, live attenuated vaccine, viral vector vaccine, protein subunit vaccine, RNA vaccine, DNA vaccine and virus-like particle (VLP) vaccine (Li et al., 2022). The immune response of human body after vaccination is acquired immunity. In the process of vaccine-induced immunity, CD4+T cells can differentiate into Th cells by TCR recognition antigen peptide-MHC class II molecular complex, CD8+T cells can differentiate into cytotoxic T cells (CTL) by TCR recognition antigen peptide-MHC class I molecular complex, and B cells are activated to produce antibodies with the help of Th cells. After antigen stimulation, B and T cells form corresponding memory cells, thus protecting the body from the invasion of the same pathogen. Now WHO has designated five VOCs (variants of concern), including Alpha (B.1.1.7), Beta (B.1.351), Gamma (P.1), Delta (B.1.617.2) and Omicron (B.1.1.529). At present, Omicron is considered to be the main VOCs in circulation in the world (Karim and Karim, 2021; Cele et al., 2022; Suryawanshi et al., 2022). With the continuous emergence of virus variation and immune escape, the serum neutralization titer of people vaccinated or even enhanced vaccination was generally decreased compared with the neutralization titer of the original strain, among which the sensitivity of Beta and Omicron was the highest, and the risk of infection with Beta, Gamma, Delta and Omicron variants was higher than that of Alpha variants (Bates et al., 2021; Caniels et al., 2021; Liu et al., 2021). The booster dose of each vaccine could significantly improve the neutralization ability of the vaccine serum to VOCs, but the Omicron variants still showed obvious evasion from vaccine serum, and the protective effect decreased significantly with the passage of time (Garcia-Beltran et al., 2022; Hoffmann et al., 2022). Although vaccines can indeed reduce the risk of infection and mortality of patients, the main problems of vaccines are the decrease of neutralization activity of mutants and adverse events related to vaccination. Adopting mix-and-match vaccines and developing new vaccines are still the main directions to overcome the epidemic situation of COVID-19 in the future.

The COVID-19 pandemic has caused irreparable damage to global health and the economy, and the crisis has not yet been brought under control. Tens of thousands of people have lost their lives due to the strong infectivity and pathogenicity of the virus itself and the short-term lack of medical equipment. Although several candidate vaccines have been developed for COVID-19 and the global vaccination program has brought hope for COVID-19 control, there are still great challenges regarding the emergence of mutants and immune escape of pathogens. Therefore, we continue to learn more about the immune mechanism of SARS-CoV-2 to find more cost-effective therapeutic products, which is crucial to the fight against this pandemic.

In the innate immune system, a series of transmission pathways and inflammatory receptors, including TLRs, RLRs, NLRs and cGAS-STING, play an indispensable role in inhibiting virus replication and controlling virus transmission. Downstream, the production of interferon signals and cytokines and cell death can also reduce virus replication and eliminate infected cells, which is characteristic of innate immunity. On the other hand, abnormally activated pathways may have serious negative effects, resulting in cytokine storms related to acute and critical illness and death. Cytokine storms play a key role in tissue injury and multiple organ failure. We review the cascade release of cytokines in the positive feedback loop, the molecular mechanism of innate immune-mediated cell death and the related pathological processes. How to regulate the rampant inflammatory response and cell death and achieve a balance between inflammation and immune regulation to help the body effectively eliminate pathogens remains an important research question. The adaptive immune response is also important for the control and elimination of pathogenic viruses. The key to the successful development of vaccines is to accurately understand the immune memory induced by adaptive immunity and related virus infection. To further understand the adaptive immune response in COVID-19, it is necessary to study the combined processes of antigen-specific CD4+ cells, CD8+ cells and antibodies in critically ill patients. Studies with longer follow up and larger cohorts may be needed to study the relationships between cross-reactive immune responses, postinfection immune memory, changes in viral load and infection duration.

## Author contributions

ZR, GC, ZY conceptualized the review. YS conducted an online research to find the latest related publications and wrote this review, HW, YZrevised, edited the draft of manuscript. All authors performed literature reviews, synthesized the information and approved the manuscript.

## Funding

This study was sponsored by grants from National Key Research and Development Program of China (2018YFC2000500), National Natural Science Foundation of China (U2004121, 82070643 and U1904164), Research Project of Jinan Microecological Biomedicine Shandong Laboratory (JNL-2022015B).

## Conflict of interest

The authors declare that the research was conducted in the absence of any commercial or financial relationships that could be construed as a potential conflict of interest.

## Publisher’s note

All claims expressed in this article are solely those of the authors and do not necessarily represent those of their affiliated organizations, or those of the publisher, the editors and the reviewers. Any product that may be evaluated in this article, or claim that may be made by its manufacturer, is not guaranteed or endorsed by the publisher.
